# Early Recognition of Immune Reconstitution Inflammatory Syndrome Leads to Avoidance of Endotracheal Intubation

**DOI:** 10.4021/wjon527w

**Published:** 2012-08-26

**Authors:** Johan Barretto, Baldeep Wirk

**Affiliations:** a University of Florida, Department of Medicine, College of Medicine USA; b University of Florida, Division of Hematology-Oncology, College of Medicine, USA

**Keywords:** Immune reconstitution inflammatory syndrome, IRIS, Neutrophil recovery, Chemotherapy

## Abstract

Immune reconstitution inflammatory syndrome occurs in patients with rapidly recovering immune systems in response to antigens (viable pathogens, nonviable pathogen debris, host antigens or tumor antigens). The acronym IRIS, Greek for spectrum of color, is often used for immune reconstitution inflammatory response syndrome and reflects the wide spectrum of clinical manifestations associated with this entity. This is a case report of an acute myelogenous leukemia patient with neutropenia after cytotoxic chemotherapy who developed severe dyspnea and new pulmonary infiltrates temporally associated with rapid neutrophil recovery. The incidence, pathogenesis, clinical presentation and therapy of IRIS will be discussed in this article. There should be an increased awareness of the many clinical manifestations of IRIS in hematologic malignancy patients with rapid neutrophil recovery after cytotoxic chemotherapy, in order to allow prompt institution of corticosteroids which could be life saving.

## Introduction

Immune reconstitution inflammatory syndrome is also known by the acronym IRIS, Greek for spectrum of color. Indeed IRIS has a wide spectrum of clinical manifestations. IRIS is an inflammatory response to antigens (viable pathogens, nonviable pathogen debris, host antigens or tumor antigens) in patients with rapidly recovering immune systems [1]. IRIS can be unmasking of covert infections in immunosuppressed hosts who cannot mount an immune response to viable pathogens until immune system recovery. In paradoxical IRIS, there is clinical deterioration of an infection despite effective antimicrobials and is caused by restoration of the immune response to antigens from dying nonviable pathogens [1]. This article highlights the importance of maintaining a high index of suspicion for the immune reconstitution inflammatory syndrome in hematologic malignancy patients with rapid neutrophil recovery after cytotoxic chemotherapy. This could avoid erroneous changes in antimicrobial therapy and allow prompt institution of corticosteroids which could be life saving.

## Case Report

A 56 years old Caucasian female with no prior medical history developed acute myelogenous leukemia (AML), CD13+, CD33+, CD11+, cytogenetics 46 XX, with FMS-like tyrosine kinase 3-internal tandem duplication and nucleophosmin 1 mutation. Although she had primary induction failure to idarubicin and cytarabine (ara-c), she had complete remission to fludarabine, ara-c, idarubicin with granulocyte colony stimulating factor (FLAG-I). Then FLAG-I consolidation was given. Neutropenic fever (39 °C) developed 11 days after start of consolidation chemotherapy when the neutrophil count was zero. Cefepime was begun. Blood cultures showed vancomycin resistant enterococci faecium (VRE) sensitive to linezolid and this was added. Fluconazole 400 mg a day and acyclovir 800 mg twice a day as neutropenic prophylaxis were given. Serum galactomannan index (GMI), fungal blood cultures and urine cultures were negative. Chest computerized tomography (CT) was unremarkable. The central line was removed and the VRE bacteremia cleared. Fever resolved. Transesophageal echocardiogram showed no vegetations. Renal and liver function tests were normal. Granulocyte colony stimulating factor (GCSF) was continued. Neutropenia resolved 15 days after start of consolidation chemotherapy when the absolute neutrophil count (ANC) was 850/microliter. GCSF was discontinued on day 16 when the white blood cell count was 4200/microliter, neutrophil count 1800/microliter, hemoglobin 10.3 g/dL, platelets 46,000/microliter. The ANC rose to 4800/microliter within 4 days from a nadir of zero.

**Figure 1:**
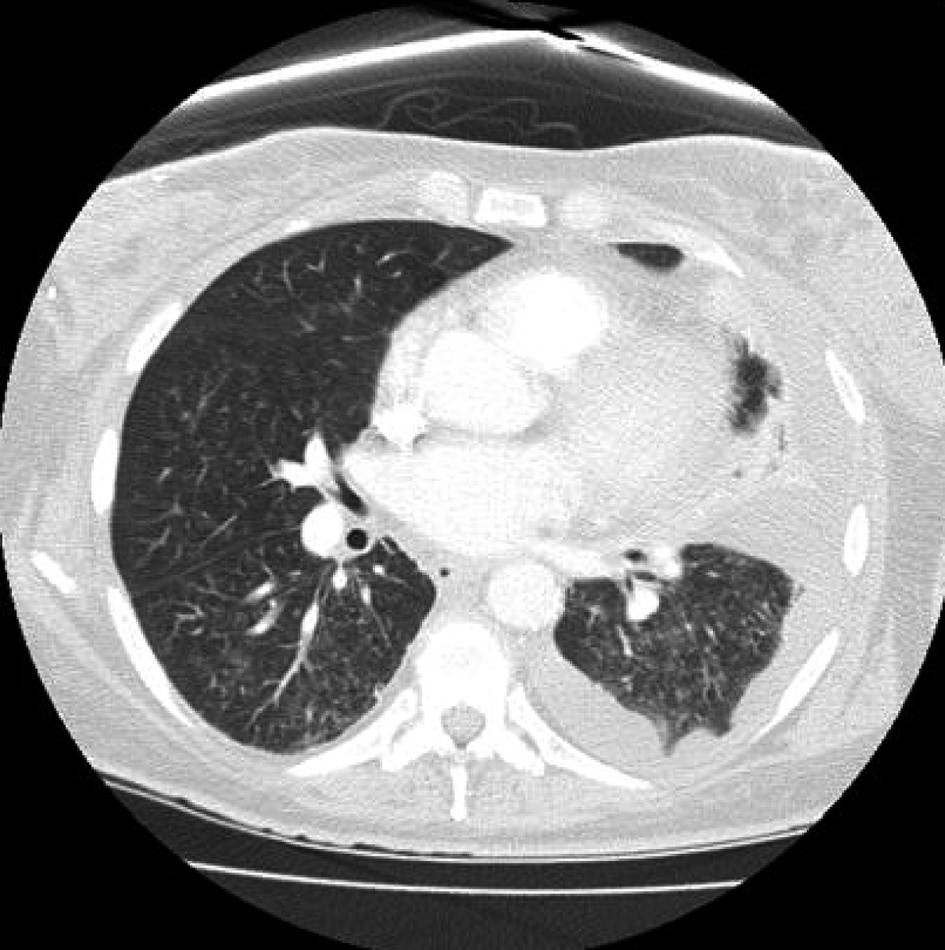
Sudden onset of diffuse interstitial infiltrates and left sided pleural effusion.

**Figure 2:**
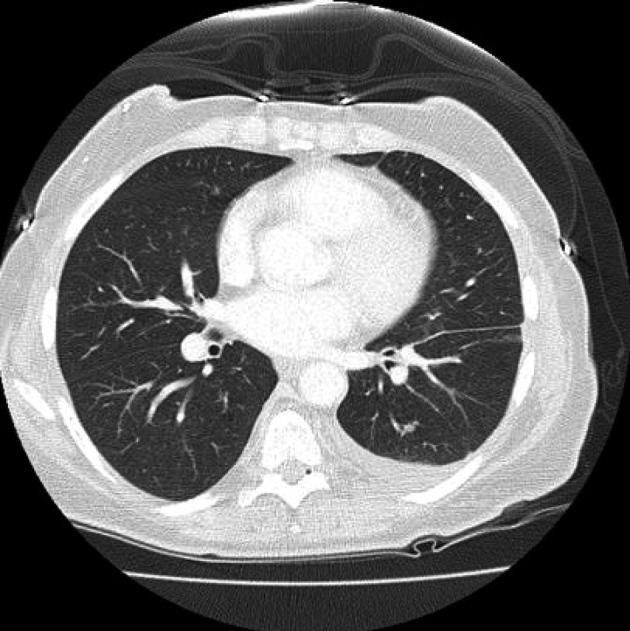
Prompt resolution of infiltrates and pleural effusion after start of corticosteroids.

On day 16 after consolidation, sudden onset of dyspnea (PO2 60 mmHg, oxygen saturation 80%, PCO2 57 mmHg) and fever (38.3 degrees Celsius) developed. Blood pressure was 125/68, heart rate 80 beats/minute, and respirations 18/minute. No recent blood transfusions had been given for 3 days. Chest CT showed diffuse interstitial infiltrates and a new left sided pleural effusion suggestive of atypical pneumonia (Fig. 1). CT abdomen showed no hepatosplenic lesions. Cefepime, linezolid were continued and azithromycin added. After intensive care unit (ICU) transfer, non-invasive positive pressure ventilation (BIPAP) was begun. Echocardiogram showed left ventricular ejection fraction 55-60%. Bronchoscopy and bronchoalveolar lavage (BAL) showed no growth on bacterial, fungal or mycobacterial cultures. Serum cytomegalovirus deoxyribonucleic acid polymerase chain reaction (CMV DNA PCR) and adenovirus DNA PCR were negative. BAL was negative for respiratory viruses by PCR (influenza A and B, respiratory syncytial virus, parainfluenza virus, human metapneumonovirus, rhinovirus, and adenovirus). Transbronchial biopsy was not done due to thrombocytopenia. Aerobic, anaerobic, fungal and mycobacterial blood and sputum cultures were negative. Diagnostic thoracentesis of a 100 mL was transudative with negative bacterial, fungal and mycobacterial cultures. Gomori methenamine silver stain, acid fast bacilli stain and Fite stain were negative on the BAL and pleural fluid and there was no evidence of *Pneumocystis jirovecii* or nocardiosis. Furosemide had no response. Given the negative infectious workup and the correlation of her acute respiratory symptoms with neutrophil recovery, methylprednisolone 2 mg/kg/ day was given for immune reconstitution inflammatory syndrome (IRIS). She improved dramatically in 24 hours. BIPAP was discontinued. She was discharged from the ICU the next day and went home 4 days later on a tapering course of prednisone without the need for supplemental oxygen. Chest CT showed good resolution of the pleural effusion and interstitial infiltrates (Fig. 2). Prompt identification of IRIS and initiation of corticosteroids led to avoidance of mechanical ventilation and possibly a prolonged ICU course.

## Discussion

### Incidence of iris

Human immunodeficiency virus (HIV) patients given antiretroviral therapy have a 10-25% incidence of IRIS due to recovery of CD4+ lymphocyte levels and adaptive immunity [2]. IRIS also occurs in other populations with rapidly recovering immune systems such as neutropenic hematological malignancy patients after neutrophil recovery, solid organ and hematopoietic cell transplant recipients after immunosuppression withdrawal, post partum women, and with cessation of tumor necrosis factor-alpha (TNF-α) antagonist therapy [3]. Pathogenesis of IRIS is poorly understood (Table 1). Possible mechanisms include host immunity restoration during effective antimicrobial therapy which potentiates a shift from dominant T helper (Th) responses that are anti- inflammatory (Th2 and regulatory T cells) to proinflammatory T cells (Th1 and Th17) [3]. Genetic predisposition with distinct human leukocyte antigen (HLA) profiles and gene polymorphisms of cytokines such as IL-6 and TNF-α have been implicated [4]. For example, HIV CMV retinitis or encephalitis IRIS was associated with human leukocyte antigen HLA A2, B44, and DR4 [4, 5]. Our patient did not have these HLA antigens. Elevation of serum interleukin (IL)-6 has been seen during IRIS irrespective of the provoking pathogen [5]. Leukotrienes, released by mast cells, may be involved in the pathogenesis of IRIS. IRIS associated with urticarial vasculitis and tuberculosis was treated with the leukotriene receptor antagonist monteleukast successfully [6]. IRIS has been linked with mycobacterial, viral and fungal organisms such as *Mycobacterium avium, leprae* or *tuberculosis*, cytomegalovirus, disseminated candidiasis, invasive aspergillosis, cryptococcosis, histoplasmosis and *Pneumocystitis jirovecii* [2].

**Table 1:** Pathogenesis of Immune Reconstitution Inflammatory Syndrome and Potential Therapeutic Approaches

Category	Mechanism	Therapy
Proliferation of Th1 and Th17 cells	Proinflammatory. Activate macrophages. Promote NK cell cytotoxicity. Cause IRIS. Involved in solid organ transplant rejection.	Corticosteroids (decrease Th1). Statins (decrease Th1 and Th17). Infliximab for steroid refractory cases (TNF-α antagonist which inhibits macrophages).
Suppression of Th2 and regulatory T cells	Usually are anti-inflammatory and leads to tolerance but reduction in levels in IRIS potentiates inflammation.	Corticosteroids (increase Th2 and regulatory T cells). Statins (increase Th2 and regulatory T cell responses).
Leukotrienes	Produced by mast cells. Proinflammatory.	Monteleukast
Genetic predisposition	Ancestral haplotype HLA A2, B44, DR4. Regulatory cytokine gene polymorphisms of IL-6 and TNF-α	N/A
Unmasking IRIS	Immune response to covert infection (viable pathogen antigens) such as chronic disseminated candidiasis in neutropenic hematological malignancy patients after neutrophil recovery.	Antifungals. Corticosteroids.
Paradoxical IRIS	Immune response to nonviable dead pathogen debris, tumor antigens or host antigens. For example pulmonary IRIS after neutrophil recovery in effectively treated invasive pulmonary aspergillosis with decrease in serum GMI.	Corticosteroids
Natural killer cells	Activating KIRs encoded by KIR genes 3DS1 and 2DS5 more common in patients with IRIS.	N/A

Abbreviations: NK: natural killer cells; Th: helper T cells; IRIS: immune reconstitution inflammatory syndrome; TNF-α: tumor necrosis factor alpha; GMI: galactomannan index; KIRs: killer immunoglobulin like receptors; N/A: non-applicable.

### Clinical presentation

Neutropenic hematologic malignancy patients undergoing effective antifungal therapy for invasive pulmonary aspergillosis (IPA) had a 4 fold increase in the mean volume of lung nodules in the first week with neutrophil recovery, associated with cavitation and the air crescent sign [7]. This is related to recovery of innate immunity and the lung damage is due to release of neutrophil proteases [7]. Patients with cavitation had better outcomes [7]. Neutrophil recovery in hematologic malignancy patients after cytotoxic chemotherapy can also lead to IRIS [8]. Nineteen hematological malignancy patients with neutropenia and proven/probable IPA, developed clinical (dyspnea, hypoxia with need for oxygen or mechanical ventilation, hemoptysis, chest pain) and radiological deterioration (pulmonary infiltrates, pleural effusions, lymphadenopathy, cavitation) associated with neutrophil recovery despite improving sequential serum GMI titers indicating treated IPA [8]. Onset of IRIS was at a mean of 2 days (range -8 to +15 days) from resolution of neutropenia to an absolute neutrophil count of > 500 /microliter [8]. In cases of IPA related IRIS, the rapidity of neutrophil recovery has been correlated with the degree of associated morbidity. In an Italian study of 20 neutropenic hematologic malignancy patients, the risk of pulmonary IRIS was higher when the ANC recovered from less than 100/microliter to greater than 4500/per microliter within a 5 day period as in our case [9]. The risk of developing IRIS was 75% with rapid ANC recovery versus 17% with more gradual recovery [5].

Undetected chronic disseminated candidiasis in neutropenic hematologic malignancy patients becomes apparent as fever, abdominal pain and hepatosplenic lesions with neutrophil recovery and is a manifestation of IRIS. Liver biopsy shows granulomas with T-cell infiltration and negative cultures [10]. In 10 patients with persistent chronic disseminated candidiasis despite 34 days of antifungal therapy, only corticosteroids resulted in resolution of fever and abdominal pain after a median of 4 days [11].

### Therapy

IRIS therapy is based on case reports and small series. Prognosis of IRIS after neutrophil recovery in cancer patients is generally good, much like HIV IRIS which heralds immune reconstitution with diminishing HIV viral loads and better survival [8]. However, some patients with pulmonary IRIS develop respiratory failure and may not survive [8]. After excluding other infections, methylprednisolone 2 mg/kg/d intravenously for 3 - 7 days has been used successfully in IPA patients with IRIS and impending respiratory failure lending further support to the immune etiology of IRIS [8]. Corticosteroids reduce Th1 and expand anti-inflammatory Th2 and regulatory T cell populations [2]. A therapeutic benefit of TNF-α inhibitors has been seen in steroid refractory cases [12]. A patient with miliary tuberculosis treated effectively with antimicrobials, developed paradoxical brain and lymph nodes lesions that were refractory to prolonged high dose corticosteroids but responded to infliximab [12]. TNF-α activates macrophages which allow granuloma formation. TNF-α inhibition with infliximab contributed to the resolution of the neurologic symptoms, brain lesions and lymphadenopathy, which were biopsy proven granulomas, culture negative for *Mycobacteria tuberculosis* (paradoxical IRIS) [12]. Statins potentiate anti-inflammatory T helper responses (Th2 and regulatory T cells), reduce proinflammatory T cells (Th1 and Th17), diminish tissue neutrophil migration and could be useful in treating IRIS [13, 14]. Case reports have also shown leukotriene inhibitors (monteleukast) to be beneficial in treating IRIS [6].

### Conclusions

No specific biomarkers to distinguish IRIS from progressive infection exist, making the timely diagnosis of lRIS challenging. Diagnosis of IRIS relies mainly on clinical criteria, such as temporal association of rapid neutrophil recovery with clinical and radiologic deterioration and exclusion of other causes such as infection (Table 2). In our patient, dyspnea and pulmonary infiltrates developed temporally associated with rapid neutrophil recovery (from ANC 0 to 4800/microliter in 4 days). After excluding infections, high dose corticosteroids were given to successfully avert respiratory failure. The etiology of pulmonary IRIS in our patient is unclear but may have been related to dying pathogen debris or tumor antigens. In hematologic malignancy patients with rapid neutrophil recovery after cytotoxic chemotherapy, maintaining a high index of suspicion for IRIS could avoid erroneous changes in antimicrobial therapy and allow prompt institution of corticosteroids which could be life saving.

**Table 2:** When to Suspect Immune Reconstitution Syndrome in Hematologic Malignancy Patients Undergoing Cytotoxic Chemotherapy

Clinical Criteria	Laboratory Values
Temporal association with rapid onset of neutrophil recovery	absolute neutrophil count recovery < 100/microliter to > 4500/microliter within 5 days
Paradoxical onset of clinical and radiologic deterioration despite effective antimicrobial therapy	Exclusion of other causes. All cultures negative (blood, urine, sputum, bronchoalveolar lavage) and/or > 50% decrease in sequential serum galactomannan titers
Unmasking of covert infection (fever, abdominal pain)	New hepatosplenic lesions seen after neutrophil recovery, culture negative with liver biopsy showing granulomas with T cell infiltration (chronic disseminated candidiasis)
Granuloma formation in affected organ	Relies on Th 1 cytokines and 1 alpha-hydroxylase of activated macrophages increases synthesis of 1, 25 (OH)_2_ vitamin D_3_ causing hypercalcemia
